# Peripheral Effects of Nesfatin-1 on Glucose Homeostasis

**DOI:** 10.1371/journal.pone.0071513

**Published:** 2013-08-15

**Authors:** Ziru Li, Ling Gao, Hong Tang, Yue Yin, Xinxin Xiang, Yin Li, Jing Zhao, Michael Mulholland, Weizhen Zhang

**Affiliations:** 1 Department of Physiology and Pathophysiology, Peking University Health Science Center, Beijing, China; 2 College of Binzhou Polytechnic, Binzhou, Shandong Province, China; 3 Department of Surgery, University of Michigan Medical Center, Ann Arbor, Michigan, United States of America; Tohoku University, Japan

## Abstract

**Aims/hypothesis:**

The actions of peripherally administered nesfatin-1 on glucose homeostasis remain controversial. The aim of this study was to characterize the mechanisms by which peripheral nesfatin-1 regulates glucose metabolism.

**Methods:**

The effects of nesfatin-1 on glucose metabolism were examined in mice by continuous infusion of the peptide via osmotic pumps. Changes in AKT phosphorylation and Glut4 were investigated by Western blotting and immnuofluorescent staining. Primary myocytes, adipocytes and hepatocytes were isolated from male mice.

**Results:**

Continuous peripheral infusion of nesfatin-1 altered glucose tolerance and insulin sensitivity in mice fed either normal or high fat diet**,** while central administration of nesfatin-1 demonstrated no effect. Nesfatin-1 increases insulin secretion in vivo, and in vitro in cultured min6 cells. In addition, nesfatin-1 up-regulates the phosphorylation of AKT in pancreas and min6 islet cells. In mice fed normal diet, peripheral nesfatin-1 significantly increased insulin-stimulated phosphorylation of AKT in skeletal muscle, adipose tissue and liver; similar effects were observed in skeletal muscle and adipose tissue in mice fed high fat diet. At basal conditions and after insulin stimulation, peripheral nesfatin-1 markedly increased GLUT4 membrane translocation in skeletal muscle and adipose tissue in mice fed either diet. In vitro studies showed that nesfatin-1 increased both basal and insulin-stimulated levels of AKT phosphorylation in cells derived from skeletal muscle, adipose tissue and liver.

**Conclusions:**

Our studies demonstrate that nesfatin-1 alters glucose metabolism by mechanisms which increase insulin secretion and insulin sensitivity via altering AKT phosphorylation and GLUT 4 membrane translocation in the skeletal muscle, adipose tissue and liver.

## Introduction

Obesity has become a medical and economic burden worldwide. Obesity causes impaired glucose tolerance and insulin resistance, often leading to type 2 diabetes [1], [2]. The onset of type 2 diabetes typically follows a phase of altered glucose metabolism typified by insulin resistance, abnormal fasting glucose levels, and impaired glucose tolerance. Management of glucose intolerance is often difficult, in part because of incomplete understanding of the mechanisms by which over-nutrition impairs glucose homeostasis.

Contemporary studies have demonstrated that hypothalamic nuclei in the central nervous system are major sites which sense overall energy balance and affect metabolic rate and glucose homeostasis [3], [4]. Recent studies indicate that gastric endocrine cells participate in these functions [5]. Gastric X/A-like endocrine cells synthesize and secrete ghrelin and nesfatin-1 which act as orexigenic and anorexigenic hormones, respectively [6]. While a number of studies have focused on ghrelin in control of appetite and glucose metabolism through effects on hypothalamic neurons, little is known of the mechanisms by which nesfatin-1 impacts glucose homeostasis.

Nesfatin-1, an 82 amino acids peptide, was originally identified in the hypothalamus as the N-terminal product of the precursor protein nucleobindin 2 protein (NUCB2) [7]. Subsequent studies confirmed expression of nesfatin-1 in the gastric mucosa [8] and the presence of circulating hormone [9]. Interestingly, ghrelin and nesfatin-1 immunoreactivities co-localize in oxyntic endocrine X/A-like cells, but in distinct subsets of vesicles [8]. Expression of nesfatin-1 mRNA has been demonstrated to be 10-fold higher in gastric mucosa than in the brain, suggesting the stomach as the main source of circulating nesfatin-1 [8]. Nesfatin-1 has been reported to cross the brain-blood-barrier via a non-saturable mechanism, providing the possibility that nesfatin-1 released from the stomach may act centrally [9], [10].

Recent observations also suggest a potential action of peripheral nesfatin-1 in control of glucose homeostasis [11]. Plasma nesfatin-1 concentrations are inversely correlated with glucose levels in rats and diabetic humans [12]. Nesfatin-1 increases glucose-stimulated insulin release from pancreatic β-cells by a direct action likely involving Ca^2+^ influx through L-type calcium channels [13]. The current study was designed to investigate the effects of peripheral nesfatin-1 on glucose homeostasis in mice fed normal and high fat diet. Peripheral infusion of nesfatin-1 significantly increased insulin secretion, altered insulin sensitivity and increased glucose disposal in insulin targeted organs, including skeletal muscle, adipose tissue and liver.

## Materials and Methods

### Materials

Phospho-AKT (ser473) and AKT rabbit monoclonal antibodies, and mouse anti-GLUT4 antibody were purchased from Cell Signaling Technology (Beverly, MA). IRDye-conjugated affinity purified anti-rabbit IgGs were purchased from Rockland (Gilbertsville, PA). Goat anti-mouse Texas Red-conjugated IgG was purchased from Santa Cruz Biotechnology (Santa Cruz, CA). Nesfatin-1 (1–82) was purchased from Phoenix Pharmaceuticals (Burlingame, CA). Alzet micro-osmotic pumps (1002) were from DURECT Corporation (Cupertino, CA). Aprotinin was purchased from Amersham Biosciences (Pittsburgh, PA). Acylated ghrelin and insulin radioimmunoassay kits were purchased from Linco Bioscience Institute (St. Charles, MO).

### Animals and Treatments

#### Animals

The animals used in this study were handled in accordance with the Guide for the Care and Use of Laboratory Animals published by the US National Institutes of Health (NIH publication no. 85–23, revised 1996), and all experimental protocols were approved by the Animal Care and Use Committee of Peking University. Twelve-week-old male C57BL/6J lean mice, high fat diet-induced obese mice and Sprague Dawley rats were used in the present study. Mice were housed in standard plastic rodent cages and maintained in a regulated environment (24°C, 12-h light, 12-h dark cycle with lights on at 07∶00 h). Regular chow and water were available *ad libitum* unless specified otherwise. In some experiments, nesfatin-1 (2.5 pmol/mouse/hour) was infused subcutaneously for 14 days. On day 11^th^ and 13^th^, OGTT and ITT were measured respectively.

#### Diets

Where indicated, 4-week-old mice were assigned to receive standard laboratory chow or a high-fat diet (45% fat, D12451; Research Diets, New Brunswick, NJ) for 8 weeks.

#### Surgery and implantation of osmotic minipumps

Mice were anesthetized with isoflurane and an area of the back was shaved. A 1 cm incision was made in the skin and mice were implanted subcutaneously with an Alzet® osmotic minipump (Model 1002) filled with vehicle or nesfatin-1. Before implantation, pumps were filled with the test agent and then placed in a petri dish with sterile 0.9% saline at 37°C for at least 4 h prior to implantation in order to prime the pumps.

### Third Intracerebroventricular (ICV) Cannulation

Sprague Dawley rats with a body weight of 280–300 g were anesthetized with a mixture of ketamine and xylazine (13 and 87 mg/kg body weight, respectively) and placed on a stereotaxic device with the incisor bar 3.3 mm below the interaural line according to Paxinos and Watson [14]. A stainless steel 26-gauge guide cannula was implanted into the third ventricle using the following stereotaxic coordinates: 2.2 mm posterior to the bregma, 8.2 mm ventral to the surface of the skull, and directly along the midline. The cannula was anchored to the skull with screws and dental cement. An internal cannula was placed into the guide cannula to maintain patency. Rats were allowed to recover for 1 week. Guide cannula patency was assessed by injection of 10 ng angiotensin II in 5 µl saline. Cannulas were considered patent if rats consumed at least 5 ml water within 1 hour of injection. Rats with correct third ventricular cannulation were used 5 days later.

### Glucose Tolerance Test and Insulin Tolerance Test

For oral glucose tolerance tests, C57BL/6J mice were fasted for 16 hours before gastric administration of glucose (3 g/kg body weight) by gavage. For insulin tolerance tests, C57BL/6J mice were fasted for 6 hours, followed by intraperitoneal injection of insulin at a dose of 1 IU/kg body weight. Blood was drawn from a cut at the tip of the tail at 0, 15, 30, 60, 90 and 120 min, and blood glucose concentrations were detected immediately.

### Measurements of Plasma Insulin

Blood samples from C57BL/6J mice were transcardially collected after anesthesia and immediately transferred to chilled polypropyrene tubes containing EDTA-2Na (12.5 mg/ml) and aprotinin (1000 units/ml) and centrifuged at 4°C. The plasma was separated and stored at −70°C before use. Insulin was measured using ELISA kits (Millipore biomanufacturer, Billerrica, MA) according to the manufacturer’s instructions. Anti-insulin antibody was used at final dilutions of 1/100,000. All assays were performed in duplicate.

### Cultured Cells

#### Myoblasts

Myoblasts were isolated from newborn C57BL/6J mice. Muscle fragments were prepared as 1 mm^3^ pieces. Tissue pieces were incubated with pre-warmed enzyme solution containing1.5 U/ml collagenase D, 2.4 U/ml dispase II (Boehringer Mannheim Corp.) and 2.5 mM CaCl_2_ at 37°C for 20 min and homogenized every 5 min. Cell suspension was filtered through 100-µm nylon mesh and collected into 20-ml centrifuge tubes. Supernatants were shaken and pipetted gently to further separate cells, then centrifuged at 350 g for 8–10 min. Cell pellets were re-suspended by gently pipetting in 10 ml of Ham’s F10 medium. Cells were counted with a hemocytometer, seeded in culture flasks at a density of 1.5×10^4^ cells/ml, and cultured in DMEM medium supplemented with 10% FBS at 37°C in a humidified incubator with 5% CO_2_. The culture medium was changed every 24 hours. Cultured cells were maintained for 4–6 days, then induced to differentiate with culture medium containing 2% FBS. Cell fusion and myotube formation were observed from 4–8 days.

#### Adipose cells

C57BL/6J mice were sacrificed and epididymal fat pads were harvested. Tissue was transferred to a low-density polypropylene vial and minced into pieces approximately 1 mm in diameter. Minced adipose tissues were then digested with collagenase (1 mg/ml, Invitrogen, Carlsbad, CA) in a shaking water bath at 37°C for approximate 40 min. After digestion, 3 ml of DMEM without phenol red was added to the vial and cells mixed by swirling. Cell suspension was then gently passed through a 250 µm nylon mesh filter and cells collected into 50 ml conical tubes. Cells were subsequently washed with 20 ml of DMEM without phenol red at 37°C, then allowed to precipitate for 10 min. Two hundred microliter of cell suspension was transferred into 50 ml vials with 5 ml of DMEM medium supplemented with 10% FBS, and incubated in a humid incubator at 37°C with 5% CO_2_ for 1.5 hours.

#### Hepatocytes

C57BL/6J mice were anesthetized with 1% Nembutal at a dose of 7 µl/g body weight and injected intraperitoneally with 1,000 IU heparin. After laparotomy, the portal vein was cannulated. The liver was perfused with 20 ml pre-warmed 37°C DHanks buffer, followed by 20 ml of 0.02% collagenase (Sigma-Aldrich, CO. St. Louis. MO) at a flow rate of 2 ml/min. After perfusion, liver tissues were removed and washed with 20 ml DHanks Buffer. The capsule of the liver was removed, and hepatic tissues were dispersed and incubated in 20 ml of 0.01% collagenase in a shaking water bath at 37°C for approximately 15 min. Cell suspension was then filtered through two-layers of 60–80 µm nylon mesh, centrifuged at 500 rpm and washed twice with DMEM medium to remove tissue dissociation enzymes, damaged cells, and non-parenchymal cells. Dispersed hepatocytes were counted and seeded at a concentration of 5–7×10^5^ cells/100 mm dish containing 10 ml high glucose DMEM medium supplemented with 10% FBS. Cells were cultured at 37°C in a humidified atmosphere of 5% CO_2_. Culture medium was changed daily.

### Western Blot Analysis

When indicated, C57BL/6J mice were treated with normal saline or insulin (2 U/kg body weight; Company name, City, State) via intraperitoneal injection 10 min before sacrifice of animals. Gastrocnemius muscle, adipose tissue, liver and primary cells were then isolated and homogenized in lysis buffer. Proteins were subjected to SDS-PAGE with a 10% running gel, and then transferred to a polyvinylidene fluoride membrane. Membranes were incubated for 1 hour at room temperature with 5% fat-free milk in Tris buffered saline containing Tween 20, followed by incubation overnight at 4°C with primary antibodies. Specific reaction was detected using IRDye-conjugated second antibody and visualized using the Odyssey infrared imaging system (LI-COR Biosciences, Lincoln, NE).

### Immunofluorescent Analysis

C57BL/6J mice were deeply anesthetized using pentobarbital, perfused transcardially with 20 ml 0.1 M PBS (pH 7.4), followed by 20 ml 4% paraformaldehyde in PBS. Gastrocnemius muscle and adipose tissue were quickly removed and rinsed thoroughly with PBS. The tissues were postfixed in 4% paraformaldehyde, dehydrated, embedded in wax, and sectioned at 6 µm. Paraffin-embedded sections were de-waxed, rehydrated, and rinsed in PBS. After boiling for 10 min in 0.01 mol/l sodium citrate buffer (pH 6.0), tissue sections were blocked in 5% goat pre-immune serum or 1% BSA in PBS for 1 hour at room temperature, then incubated overnight with mouse monoclonal antibody against GLUT4 (1∶100) or control IgG. Tissue sections were then incubated at room temperature for 1 hour with the following secondary antibody: goat anti-mouse Texas Red-conjugated IgG (1∶100). Controls included substituting primary antibodies with mouse IgG. Computerized image analysis (Model Leica Q550CW, Leica Qwin, Germany) was performed to quantify the immunostaining signals of cytoplasm membrane GLUT4 from mouse gastrocnemius muscle and adipose tissue.

### Statistical Analysis

All values are expressed as mean ±SEM. Statistical differences were evaluated by two-way ANOVA and Newman-Student-Keuls test. Comparisons between two groups involved use of the Student’s *t* test. *P* value <0.05 denotes statistical significance.

## Results

### Alteration of Glucose Metabolism by Peripheral Infusion of Nesfatin-1

**Figure 1:**
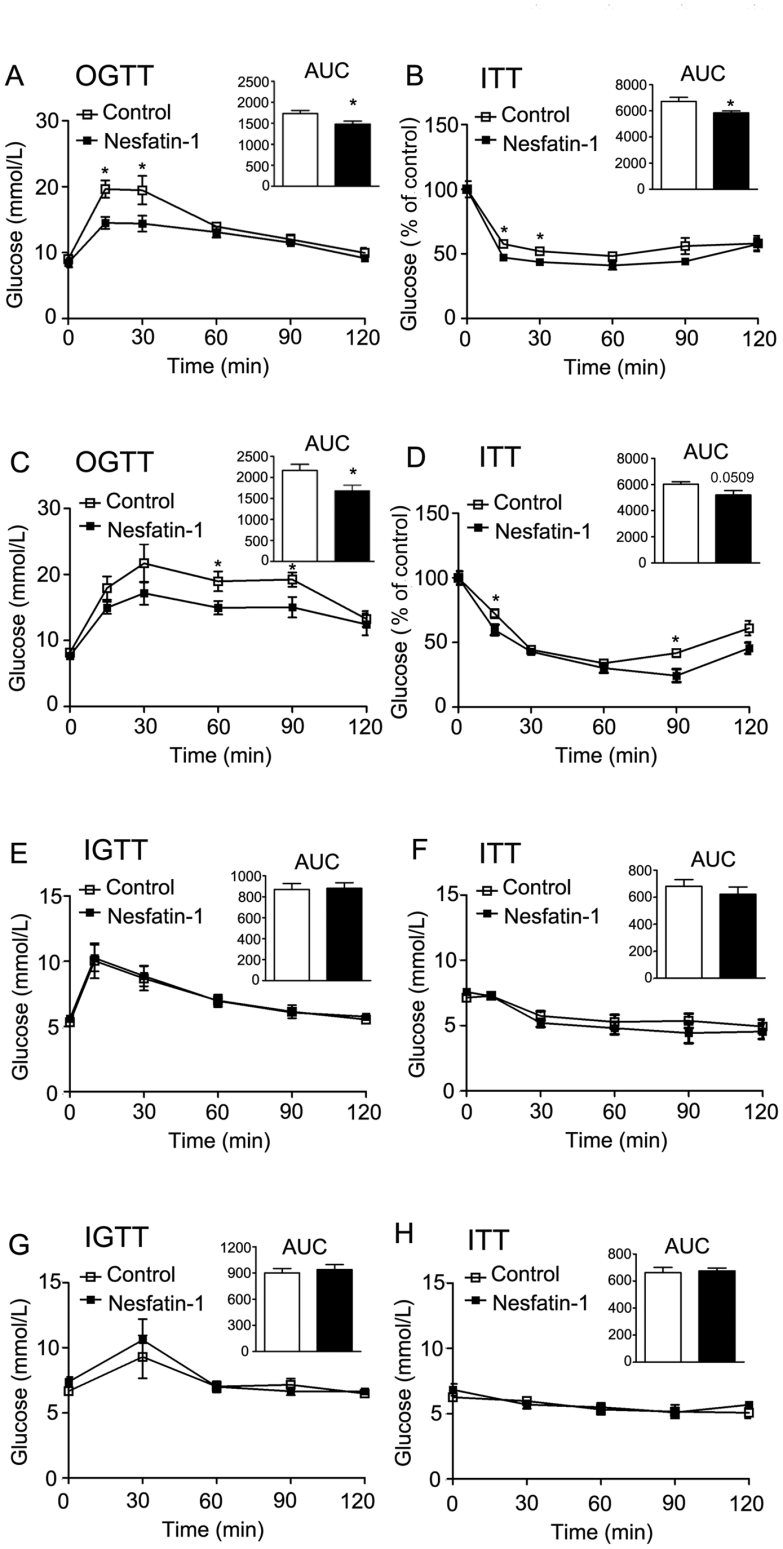
Effects of nesfatin-1 infusion on glucose homeostasis. Oral glucose tolerance tests and insulin sensitivity tests are shown in left and right panels, respectively. Results shown are for oral glucose tolerance tests (OGTT) and insulin tolerance tests (ITT) in C57BL/6J mice fed normal chow diet (A, B) or high fat diet (C, D) receiving saline (□ line) or nesfatin-1 infusion (▪ line) at a dose of 2.5 pmol/mouse/hour. Areas under curves were analyzed by Prism software. Eleven mice were examined for each condition. *P<0.05 vs. control mice. Data expressed as mean±SEM. Intraperitoneal glucose tolerance tests (IGTT) and ITT were performed in light cycle (07∶00–19∶00) (E) and dark cycle (19∶00–07∶00) (G) in rats 10 min after 3^rd^ ICV injection of nesfatin-1 or vehicle. Shown are data on IGTT and ITT obtained in light cycle (E, F) and dark cycle (G, H). Areas under curves were analyzed by Prism software. Six rats were examined for each condition. Data expressed as mean±SEM.

To determine its peripheral effect on glucose metabolism, nesfatin-1 was continuously infused at a concentration of 2.5 pmol/mouse/hour for 14 days by a subcutaneously implanted Alzet micro-osmotic pump. As shown in Figure S1A, B, D and E, peripheral infusion of nesfatin-1 had no effect on dark or light cycle food intake or body weight. In contrast, oral glucose tolerance tests (OGTT) and insulin tolerance tests (ITT) revealed that mice treated with nesfatin-1 demonstrated altered glucose metabolism and glucose disposal curves typifying higher insulin sensitivity. These alterations were observed in C57BL/6J mice fed normal chow diet (Figure 1A, B), as well as in high fat diet-induced obese mice (Figure 1C, D).

Previous studies have demonstrated that nesfatin-1 can pass through the blood-brain barrier in an unsaturated manner [9], [10]. To exclude a central effect on glucose homeostasis, nesfatin-1 was injected into the third ventricle via an implanted cannula at a dose of 0.5 µg/rat. Despite a significant reduction in dark cycle food intake after nesfatin-1 treatment (Figure S1C), central administration of nesfatin-1 demonstrated no effect on glucose tolerance and insulin sensitivity either in the dark cycle (Figure 1E, F) or the light cycle (Figure 1G, H).

### Effects of Nesfatin-1 on Islet Cell Functions

**Figure 2:**
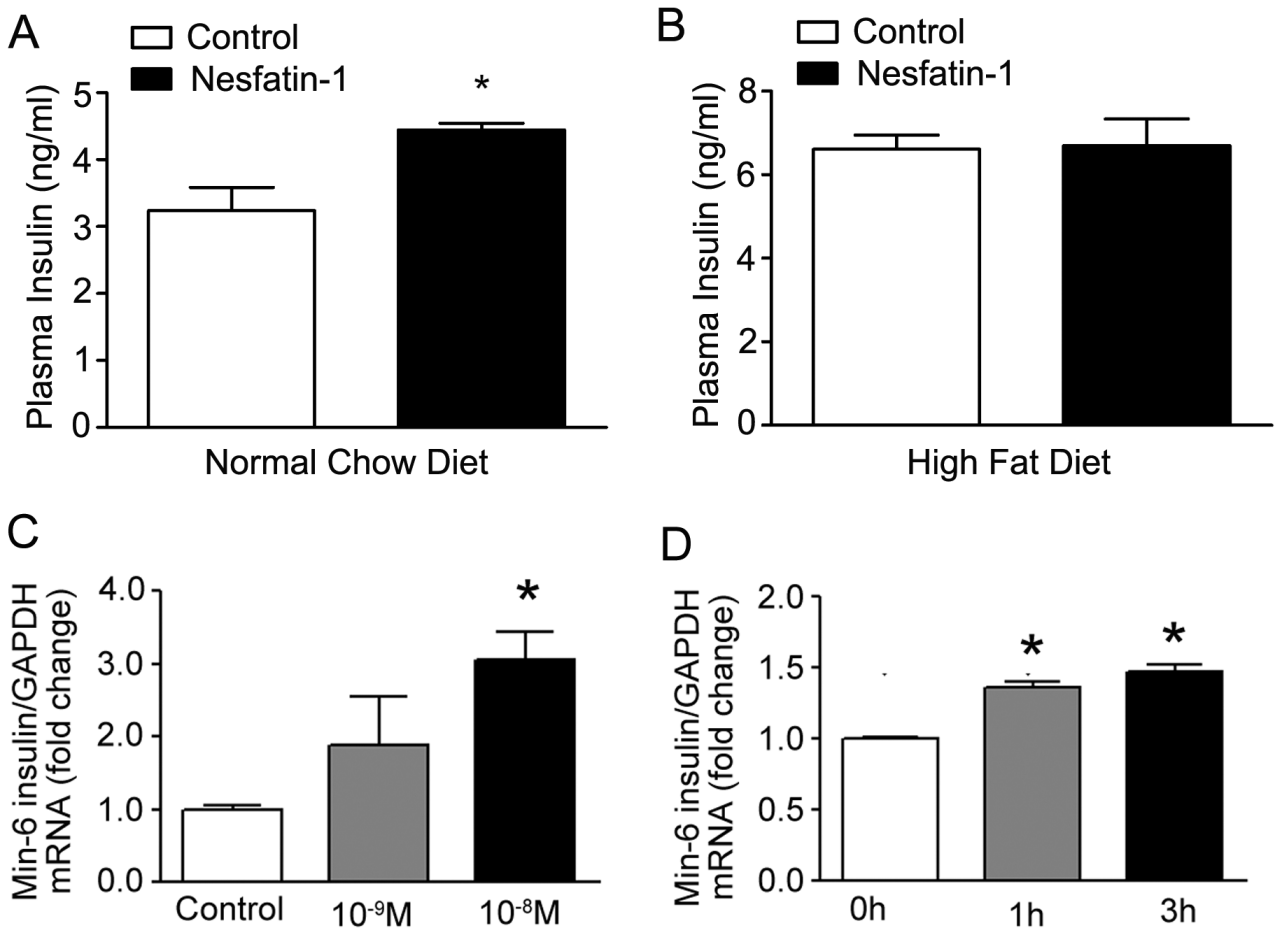
Effects of peripheral nesfatin-1 on insulin secretion. (A, B) Effects on mice. Mice were fed normal chow diets (NCD) (A) and high fat diets (HFD) (B) for 8 weeks, then received peripheral infusion of nesfatin-1 for 2 weeks. Eleven mice were examined for each condition. Data were expressed are mean±SEM; *P<0.05 vs. control mice. (C, D) Effects on min6 cells. Cultured min6 islet cells were treated with nesfatin-1 or vehicle. Insulin mRNA expression was determined by real-time RT-PCR. Data were expressed are mean±SEM; Dose-dependent (C) and time-dependent responses (D) were shown. *P<0.05 vs. control treatment.

In order to determine the mechanisms by which peripheral nesfatin-1 alters glucose metabolism, we first examined its effects on islet cell functions. As shown in Figure 2A and 2B, chronic continuous infusion of nesfatin-1 increased serum insulin concentration from 3.2±0.4 ng/ml to 4.4±0.1 ng/ml (p<0.05) in mice fed normal chow diet, while demonstrating no effect in animals fed high fat diet (6.6±0.3 vs. 6.7±0.6 ng/ml).

**Figure 3:**
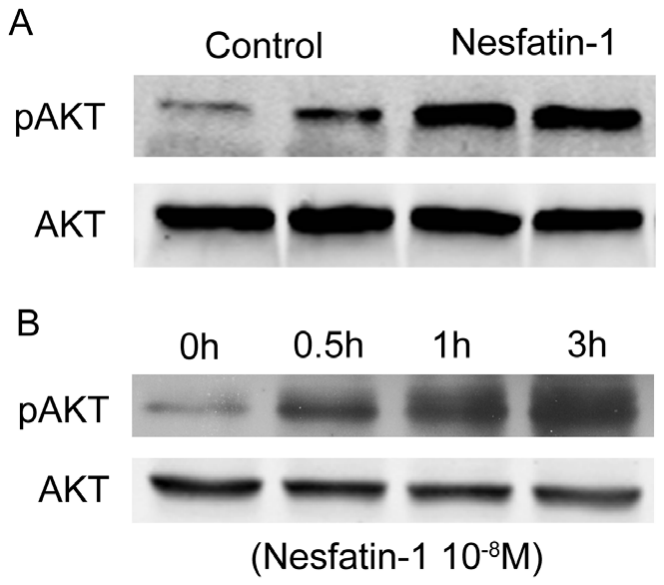
Effects of nesfatin-1 on AKT phosphorylation in pancreas (A) and min6 islet cells (B). (A) Mice were fed high fat diet for 8 weeks, then received subcutaneous infusion of saline (control) or nesfatin-1 for 2 weeks. Levels of AKT phosphorylation were examined in pancreas by Western blotting using specific antibody. Shown are representative results from 6 individual experiments. B. Cultured min6 islet cells were treated with nesfatin-1 at a dose of 10^−8^ M for the time indicated. Shown is the representative western blot from 3 separate experiments.

The direct effects of nesfatin-1 on islet cell function were further validated in cultured min6 islet cells. As shown in Figure 2C and D, nesfatin-1 markedly enhanced the insulin mRNA expression in the dose- and time-dependent manners. In addition, nesfatin-1 up-regulated the phosphorylation of AKT in pancreas (Figure 3A) and min6 islet cells (Figure 3B).

### AKT Phosphorylation and Glut 4 Translocation in Mice Fed Normal Diet

**Figure 4:**
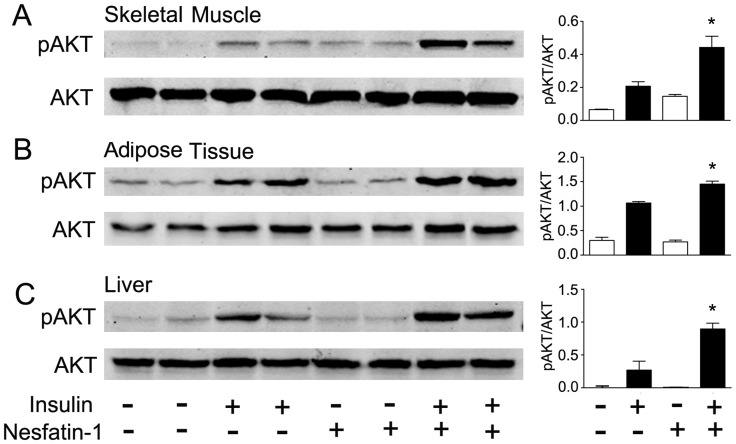
Effects of peripheral nesfatin-1 infusion on AKT phosphorylation in mice fed control diet. Mice received subcutaneous infusion of saline (control) or nesfatin-1 (2.5 pmol/mouse/hour). Insulin at a dose of 2 IU/kg was injected intraperitoneally 10 min before animals were sacrificed. Phosphorylation of AKT (ser473) was detected by Western blotting using specific antibody and normalized to total AKT. Levels of AKT phosphorylation were examined in skeletal muscle (A), adipose tissue (B) and liver (C) in mice fed normal chow diet. Intensity of phosphor-AKT signals were quantified by NIH Image J software and expressed as mean±SEM. *P<0.05 vs. insulin stimulation without nesfatin-1 treatment. Six samples were examined for each condition.

We next examined insulin sensitivity in skeletal muscle, adipose tissues and liver. We first examined the insulin signaling pathway using AKT phosphorylation as a marker. As shown in Figure 4, peripheral nesfatin-1 infusion increased phosphorylation of AKT in skeletal muscle. Upon insulin stimulation, all three tissues responded with an increased level of AKT phosphorylation. Peripheral infusion of nesfatin-1 significantly augmented phosphorylation of AKT induced by insulin in skeletal muscle, adipose tissue and liver.

**Figure 5:**
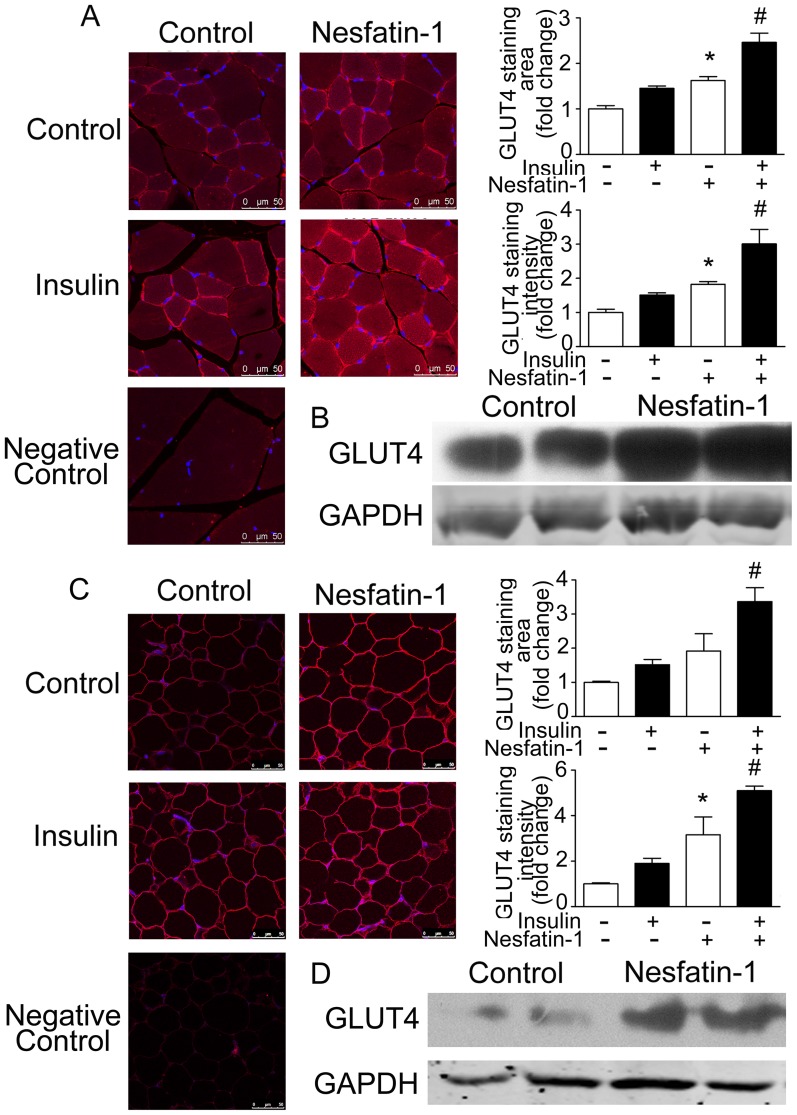
Effects of peripheral nesfatin-1 infusion on GLUT 4 in NCD mice. (A, C) Immunofluorescent staining for GLUT4. Immunofluorescent staining was performed using specific antibody against GLUT4 (red) in skeletal muscle (A) and adipose tissue (C). Nuclei were stained with Hoechst dye. Controls include substituting primary antibodies with mouse IgG. Signal intensity of cytoplasm membrane GLUT4 immunoreactivity was quantified and normalized to the basal control, Data are expressed as mean±SEM. *P<0.05 vs. control, # *P*<0.05 vs. insulin stimulation without nesfatin-1 treatment. (B, D) Western blotting for GLUT4. Western blot was performed using specific antibody against GLUT4 in skeletal muscle (B) and adipose tissue (D). GAPDH was used as the internal control. Shown were representative results from six individual experiments.

We next examined the effects of peripheral nesfatin-1 on the expression and membrane translocation of glucose transporter 4 (GLUT4), an isoform of glucose transport proteins which is expressed in adipose tissue and skeletal muscle. As shown in Figure 5, peripheral nesfatin-1 significantly increased GLUT4 membrane translocation in adipocytes and myocytes, under basal conditions and after insulin stimulation. The average area and mean intensity of GLUT4 positive immunofluorescent signals in the cytoplasmic membranes of myocytes (Figure 5A) and adipocytes (Figure 5C) were increased by peripheral nesfatin-1. In addition, significant increase in the GLUT4 expression was demonstrated in both the skeletal muscle (Figure 5B) and adipose tissue (Figure 5D).

### AKT Phosphorylation and Glut 4 Translocation in Mice Fed High Fat Diet

**Figure 6:**
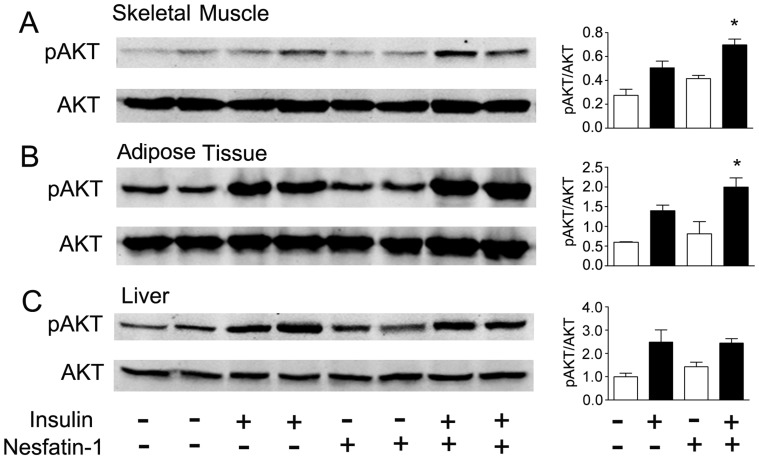
Effects of peripheral nesfatin-1 infusion on phosphorylation of AKT in mice fed high fat diet. Mice were fed high fat diet for 8 weeks, and then received subcutaneous infusion of saline (control) or nesfatin-1 for 2 weeks. Insulin at a dose of 2 IU/kg was injected intraperitoneally 10 min before animals were sacrificed. Phospho AKT (ser473) and AKT were detected by Western blotting using specific antibodies, in which AKT was used as internal controls. Shown are representative results from saline (control) and nesfatin-1 infusion mice with or without insulin injection. Levels of AKT phosphorylation were examined in skeletal muscle (A), adipose tissue (B) and liver (C) in mice fed high fat diet. Intensity of phosphor-AKT signals were quantified by NIH Image J software and expressed as mean±SEM. *P<0.05 vs. insulin stimulation without nesfatin-1 treatment. Six samples were examined for each condition.

We next examined whether peripheral infusion of nesfatin-1 alters glucose metabolism in mice fed high fat diet. Similar to observations in mice fed normal diet, peripheral nesfatin-1 demonstrated no effect on food intake and body weight in high fat diet-induced obese mice (Figure S1). As shown in Figure 6, nesfatin-1 increased AKT phosphorylation in skeletal muscle. Insulin increased AKT phosphorylation in skeletal muscle, adipose tissue and liver. Insulin-stimulated phosphorylation levels of AKT were further increased by peripheral infusion of nesfatin-1 in skeletal muscle and adipose tissue.

**Figure 7:**
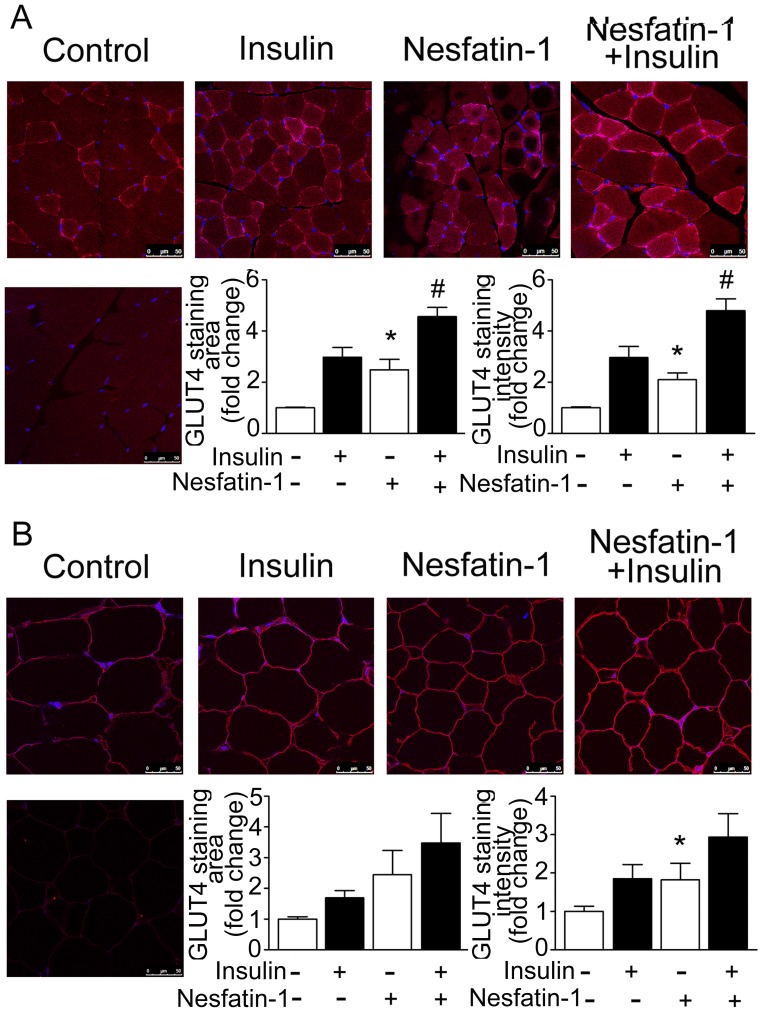
Effects of peripheral nesfatin-1 infusion on GLUT4 in mice fed high fat diet. Immunofluorescent staining was performed using specific antibody against GLUT4 (red) in skeletal muscle and adipose tissue derived from mice consuming high fat diet and either saline (control) or nesfatin-1. Nuclei were stained with Hoechst dye. Controls include substituting primary antibodies with mouse IgG. Shown were representative results from six individual experiments. Images depict immunofluorescent staining for GLUT4 (red) in skeletal muscle (A) and adipose tissue (B). Data are expressed as mean±SEM as described in Figure 4. *P<0.05 vs. control, # *P*<0.05 vs. insulin stimulation without nesfatin-1 treatment. Shown were representative results from six individual experiments.

Membrane GLUT4 in both the skeletal muscle and adipose tissue derived from mice fed a high fat diet was significantly increased by peripheral infusion of nesfatin-1 at basal conditions and after insulin stimulation. As shown in Figure 7A, average area and intensity of membrane GLUT4 immunoreactivity signals in skeletal muscle at basal conditions and after insulin stimulation were markedly increased by peripheral nesfatin-1. Similar effects were observed in adipose tissue (Figure 7B).

### Direct Effects of Peripheral Nesfatin-1 on AKT Phosphorylation

**Figure 8:**
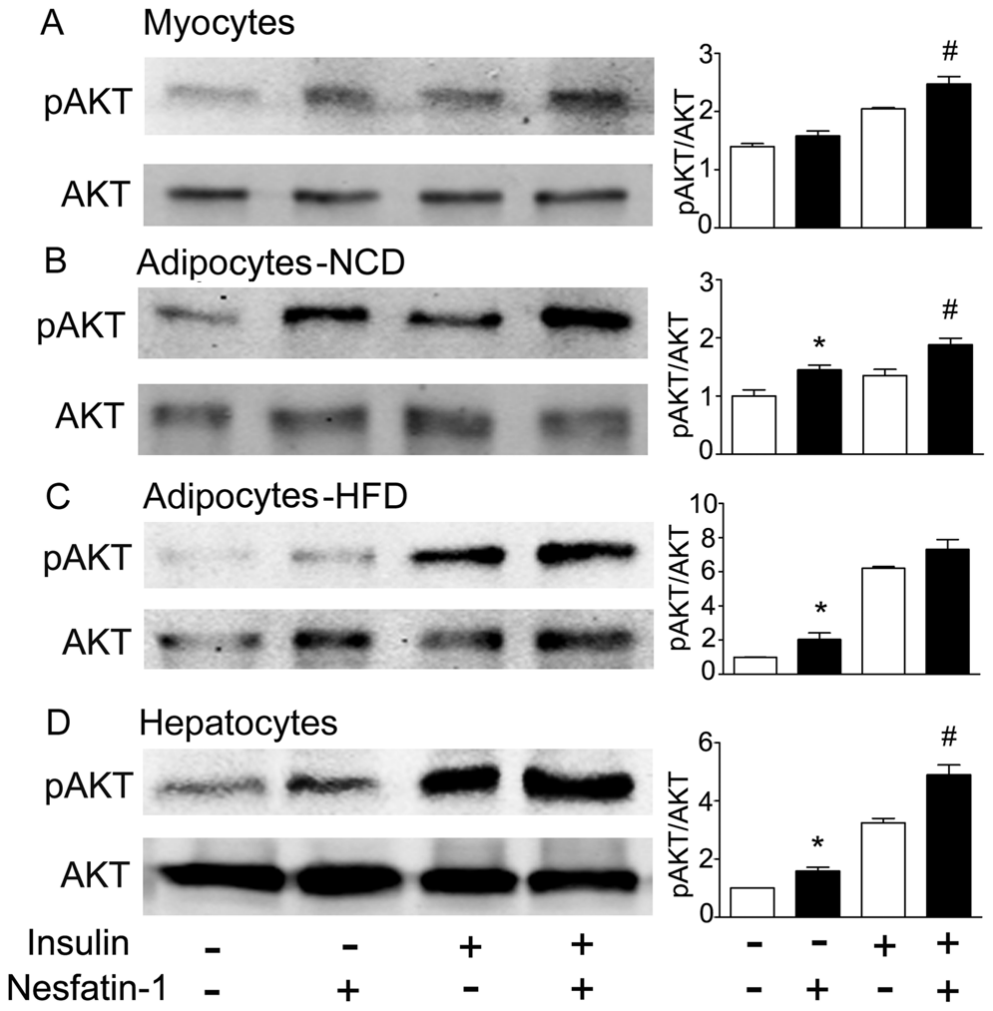
In vitro effects of nesfatin-1 on AKT phosphorylation. Cells were treated with nesfatin-1 (10^−7^ mol/L) for 30 min or/and insulin (10^−9/−10^ mol/L) 10 min before harvest of cells. Phospho AKT (ser473) and AKT were detected by Western blotting using specific antibodies, in which AKT was used as internal controls. Shown are representative results of myotubes differentiated from primary myoblasts (A), adipocytes from mice fed with NCD (B) or HFD (C) and hepatocytes (D). Levels of AKT phosphorylation are expressed as mean±SEM. *P<0.05 vs. control, # *P*<0.05 vs. insulin stimulation without nesfatin-1 treatment. Experiments were repeated more than three times for each condition.

To further determine whether the effects of peripheral nesfatin-1 are mediated directly in peripheral tissues, we next examined the effects of nesfatin-1 on phosphorylation of AKT in cells derived from insulin target organs: skeletal muscle, adipose tissue and liver. As shown in Figure 8A, nesfatin-1 at a dose of 10^−7^ mol/L stimulated phosphorylation of AKT under both basal conditions and after insulin stimulation in primary myocytes. In adipocytes isolated from mice fed with either normal chow or high fat diet, nesfatin-1 (10^−7^ mol/L) increased basal and insulin-stimulated levels of AKT phosphorylation (Figure 8B, C). In hepatocytes derived from C57BL/6J mice, the stimulatory effects of nesfatin-1 on AKT phosphorylation were detected both basally and in insulin-treated conditions (Figure 8D).

## Discussion

The major finding of this study is that peripherally administered nesfatin-1 affects glucose metabolism through actions on skeletal muscle, adipose tissue and liver. This general conclusion is supported by the following observations: 1) continuous infusion of nesfatin-1 improved glucose tolerance and insulin sensitivity in mice fed either a normal diet or a high fat diet; 2) injection of nesfatin-1 into the third ventricle demonstrated no effect on glucose homeostasis either in the light cycle or the dark cycle; 3) nesfatin-1 increased insulin secretion in vivo, and stimulated insulin mRNA expression in cultured min6 cells. In addition, nesfatin-1 up-regulated the phosphorylation of AKT in pancreas and min6 islet cells; 4) nesfatin-1 enhanced phosphorylation of AKT stimulated by insulin in skeletal muscle, adipose tissue and liver; 5) nesfatin-1 increased glucose transporter 4 (GLUT4) expression and cytoplasmic membrane translocation in the skeletal muscle and adipose tissue under both basal and insulin-stimulated conditions; 6) *in vitro* studies showed that nesfatin-1 increased both basal and insulin-stimulated levels of AKT phosphorylation in cells derived from skeletal muscle, adipose tissue and liver.

In addition to its originally identified function to inhibit dark cycle food intake, nesfatin-1 exhibits a broad array of actions, ranging from effects on glucose metabolism [11], inhibition of gastric acid secretion [15] and gastrointestinal motility [16], control of fluid and electrolyte homeostasis [17], inhibition of adipogenesis [18], counteracting NO-dependent vasodilation [19], to cardio-protection against ischemia/reperfusion injury [20]. Among these functions, the effects of nesfatin-1 on glucose homeostasis and its potential mechanisms remain controversial.

The relationship between plasma levels of nesfatin-1 and the presence of type 2 diabetes mellitus have been reported to correlate either negatively or positively. Zhang et al. reported a positive relationship between plasma nesfatin-1 levels and impaired glucose tolerance [21]. In contrast, Li et al. reported that levels of fasting plasma nesfatin-1 are lower in type 2 diabetes mellitus than in normal controls [12]. Reasons for this discrepancy remain unclear but may derive from differences in severity of disease or in different patient characteristics, for example body mass index. Studies have demonstrated elevation of nesfatin-1 expression in high fat diet-induced obese mice [22].

Conflicting results have also been reported regarding the effects of nesfatin-1 on insulin secretion. Peripheral infusion of nesfatin-1 has been reported to significantly enhance insulin secretion in rats [23], and in isolated pancreatic islets from humans [24]. The insulinotropic effect of nesfatin-1 is likely mediated by stimulation of Ca^2+^ influx through L-type Ca^2+^ channels [13]. Consistent with these reports, our studies demonstrate a moderate elevation in plasma insulin levels in response to continuous peripheral infusion of nesfatin-1 in lean mice. Furthermore, nesfatin-1 increases insulin expression and the phosphorylation of AKT in cultured min6 cells. Despite these direct effects of nesfatin-1 on islet cell functions, other mechanism such as insulin sensitivity may be involved. In the present study, insulin levels remained unaltered by nesfatin-1 in obese mice, suggesting that nesfatin-1 may alter glucose metabolism by mechanisms other than direct effects on insulin secretion. This concept is further supported by a recent study in which nesfatin-1 exhibited no effect on insulin secretion in mouse islets or cultured INS-1 cells [24].

Several observations indicate that nesfatin-1 affects glucose metabolism by increasing insulin sensitivity. A recent study by Yang et al. demonstrated that nesfatin-1 activates hypothalamic neurons in the arcuate nucleus and the paraventricular nucleus to increase insulin sensitivity in the setting of diet-induced insulin resistance by decreasing hepatic gluconeogenesis and promoting peripheral glucose uptake in skeletal muscle [25]. Studies by Gonzalez et al. indicated a tissue specific effect of nesfatin-1 on regulation of glucose metabolism: an increase in both basal and insulin-stimulated glucose uptake in adipose tissue, and a decrease in insulin-stimulated glucose uptake in L6 muscle cells [23]. Consistent with the report by Gonzalez et al., our data suggest a peripheral action of nesfatin-1 on glucose metabolism. Unlike anorexigenic effects which are mediated by a CNS mechanism, peripheral nesfatin-1 improves glucose metabolism in both lean and obese mice by direct actions on insulin target organs: skeletal muscle, adipose tissue and liver. These effects may be mediated through increased glucose transport in skeletal muscle and adipose tissue as suggested by increased GLUT4 expression and membrane translocation and insulin signaling in these tissues. These observations are congruent with previous reports [26], [27] demonstrating that GLUT4 membrane translocation in the skeletal muscle and adipose tissues is modulated by insulin signaling and therefore critical for insulin sensitivity.

There also exist conflicting data on the role of AKT signaling induced by nesfatin-1. Yang et al. reported activation of AKT by nesfatin-1 in the liver [25]. Our studies confirm this observation and extend this effect to involve skeletal muscle and adipose tissue. These results are in agreement with previous reports demonstrating that insulin-induced phosphorylation of AKT leads to GLUT4 membrane translocation and glucose uptake [28].

Both in vivo and in vitro studies demonstrate that nesfatin-1 increases phosphorylation of AKT stimulated by insulin in skeletal muscle, adipose tissue and liver in mice fed normal chow diet. This effect was detected only in skeletal muscle and adipose tissue in mice fed high fat diet. In previous studies, central administration of nesfatin-1 was able to increase insulin receptor signaling in the liver in rats fed high fat diet [25]. Our findings suggest a tissue-specific response to nesfatin-1. Similarly, differential responses to nesfatin-1 in skeletal muscle and adipose tissue have been observed by Gonzalez et al. [23]. Both glucose uptake and AKT phosphorylation in adipose tissue were significantly up-regulated by nesfatin-1. However, phosphorylation of AKT remained unchanged by nesfatin-1 despite marked increases in glucose uptake in cultured L6 myotubes.

In conclusion, our studies demonstrate that nesfatin-1 affects glucose metabolism by a direct peripheral mechanism to increase insulin secretion and insulin sensitivity via altering AKT phosphorylation and GLUT4 membrane translocation in the skeletal muscle, adipose tissue and liver.

## Supporting Information

Figure S1 **Effects of nesfatin-1 infusion on food intake and body weight.** Dark cycle (12 h) and 24 h food intake in mice fed normal chow diet (NCD) or high fat diet (HFD) during peripheral infusion of nesfatin-1 are shown in A and B, respectively. Food intake in rats before (−24 h) and after 3^rd^ ICV injection of nesfatin-1 was recorded in the dark cycle and is shown in panel C. Change of body weight after operation and final body weight are shown in panels D and E, respectively. Six mice/rats were examined for each condition. Data are expressed as mean±SEM. *P<0.05 vs. control mice.
(TIF)
