# Supplementary figures and images for: Peripheral Effects of Nesfatin-1 on Glucose Homeostasis
Source: PLoS One. 2013 Aug 15;8(8):e71513. doi: 10.1371/journal.pone.0071513 (PMC3744551; doi:10.1371/journal.pone.0071513)

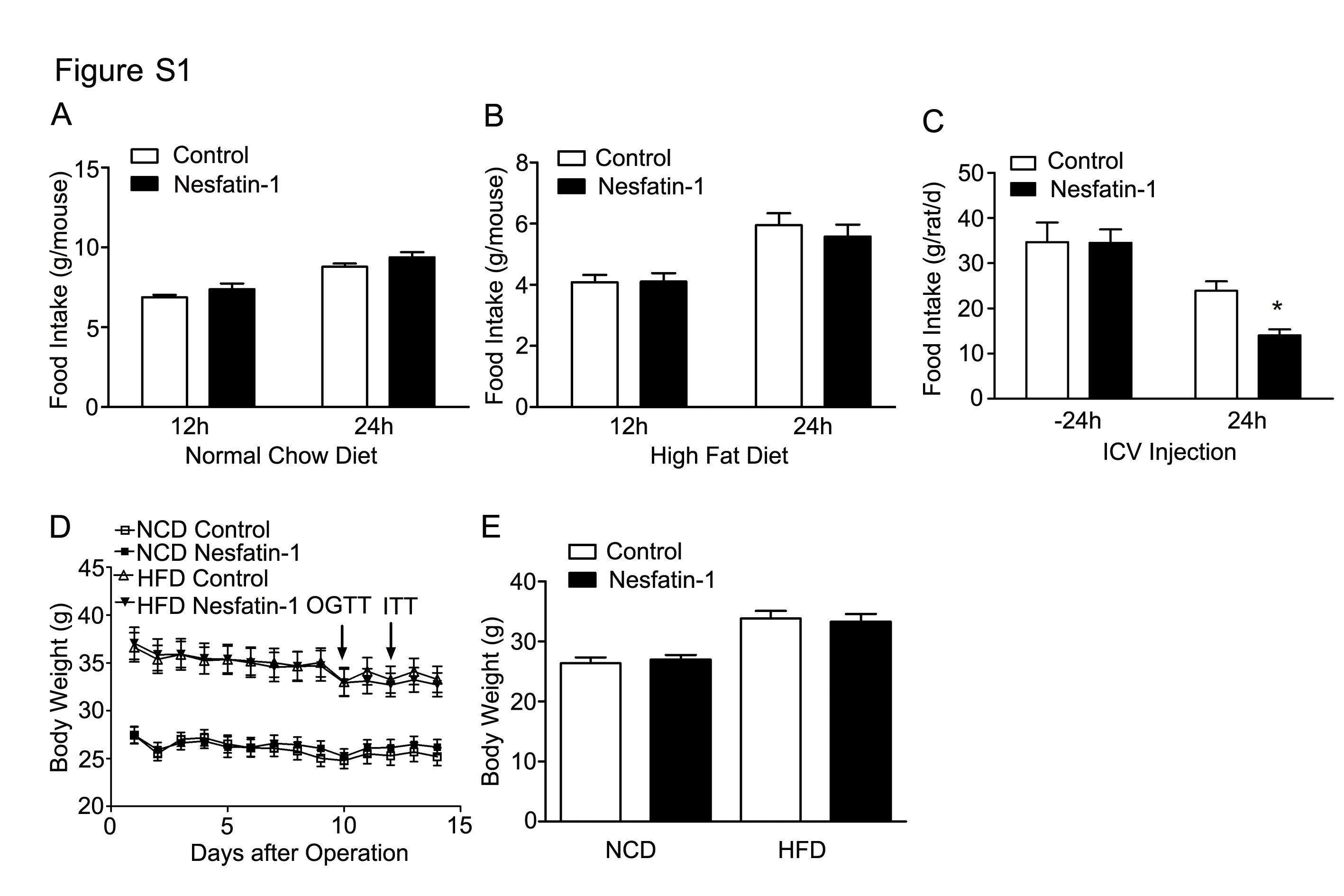

Supplement: Figure S1 — Effects of nesfatin-1 infusion on food intake and body weight. Dark cycle (12 h) and 24 h food intake in mice fed normal chow diet (NCD) or high fat diet (HFD) during peripheral infusion of nesfatin-1 are shown in A and B, respectively. Food intake in rats before (−24 h) and after 3rd ICV injection of nesfatin-1 was recorded in the dark cycle and is shown in panel C. Change of body weight after operation and final body weight are shown in panels D and E, respectively. Six mice/rats were examined for each condition. Data are expressed as mean±SEM. *P<0.05 vs. control mice. (TIF) [file pone.0071513.s001.tif]
